# Human circadian rhythm studies: Practical guidelines for inclusion/exclusion criteria and protocol

**DOI:** 10.1016/j.nbscr.2022.100080

**Published:** 2022-08-08

**Authors:** Yashar Yousefzadehfard, Bennett Wechsler, Christine DeLorenzo

**Affiliations:** a Center for Understanding Biology Using Imaging Technology, Department of Psychiatry, Stony Brook University, Stony Brook, NY, USA; b Department of Psychiatry, University of Massachusetts Medical School, Worcester, MA, USA; c Department of Psychiatry, Texas Tech University Health Science Center, Midland, TX, USA

**Keywords:** Circadian rhythm studies, Melatonin, Suprachiasmatic nucleus, Insomnia, Alcohol, Shift work

## Abstract

As interest in circadian rhythms and their effects continues to grow, there is an increasing need to perform circadian studies in humans. Although the constant routine is the gold standard for these studies, there are advantages to performing more naturalistic studies. Here, a review of protocols for such studies is provided along with sample inclusion and exclusion criteria. Sleep routines, drug use, shift work, and menstrual cycle are addressed as screening considerations. Regarding protocol, best practices for measuring melatonin, including light settings, posture, exercise, and dietary habits are described. The inclusion/exclusion recommendations and protocol guidelines are intended to reduce confounding variables in studies that do not involve the constant routine. Given practical limitations, a range of recommendations is provided from stringent to lenient. The scientific rationale behind these recommendations is discussed. However, where the science is equivocal, recommendations are based on empirical decisions made in previous studies. While not all of the recommendations listed may be practical in all research settings and with limited potential participants, the goal is to allow investigators to make well informed decisions about their screening procedures and protocol techniques and to improve rigor and reproducibility, in line with the objectives of the National Institutes of Health.

## Highlights

• This review provides critical recommendations for ensuring a rigorous circadian study.
• When available, recommendations are based on scientific rationale.
• Empirical decisions are also provided.
• The goal is to improve rigor and reproducibility of circadian studies.

## 1 Introduction

Circadian dysfunction has been implicated in diseases as distinct as cancer, obesity, and depression (Engin, 2017; Karatsoreos, 2014; Masri and Sassone-Corsi, 2018; Selvi et al., 2018). It is partly for this reason that interest in circadian rhythms on the cellular and organism-wide level has expanded greatly in the past few decades. Further highlighting its growing importance, the 2017 Nobel Prize in Physiology or Medicine was granted for work uncovering the molecular mechanisms underlying circadian regulation.

As a consequence, there is a growing need to conduct circadian studies in humans. Historically, investigators used constant routine or forced desynchrony protocols to measure the circadian phase and period, respectively. In a constant routine protocol, subjects maintain constant conditions including light, temperature and semi-recumbent posture for at least 24 h in order to study endogenous circadian rhythms without any external effects (J. E. Duffy and Dijk, 2002). In a forced desynchronization protocol, subjects are forced to an extended (28 h) or shortened (20 h) rest-activity schedule so that the possible effects of body activity rhythm on internal circadian biology can be disentangled (Strijkstra et al., 1999). However, in many situations, a more naturalistic design would be informative. The purpose of this review is to provide a guide to those wishing to produce a well-controlled, lifestyle/routine integrated circadian study in a healthy population, with recommendations that follow from the basic science and clinical literature. Studying healthy circadian rhythm physiology may also enable investigators to answer questions related to its role in pathological processes.

First, details about circadian rhythms, normal sleep architecture and terminology, as well as methods of assessment are discussed. Then, screening issues that will likely arise when selecting healthy participants for circadian studies are detailed, and finally protocol considerations are reviewed.

## 2 Literature review

Publications on Medline were searched for human clinical trials published in English examining circadian rhythms in healthy participants. Until end of April 2019 there were 585 published titles in Medline using the following search terms:

**((zeitgebers OR ”central oscillator” OR "peripheral oscillator" OR "circadian oscillator" OR “sleep complaint” OR “sleep efficiency” OR chronobiology OR “sleep deprivation” OR “constant routine” OR “phase shift” OR “circadian pace maker” OR “circadian pacemaker” OR “circadian rhythm” OR “circadian rhythms” OR “sleep time” OR “circadian phase” OR “sleep wake cycle”))**

AND.

**((“Melatonin” OR “pineal gland” OR “phase response curves” OR “suprachiasmatic nucleus”))**

After removing duplicate titles, the abstracts were thoroughly studied and those focused on healthy, non-athlete adults, without circadian complains, shift work or menstruation related sleep problems in at least one study arm were included in the review. Among largest excluded groups, 65 studies were excluded for only examining those with mental illness without a healthy control group, 92 for only examining pre-existing circadian impairment, 22 for only athletes and 33 for only pediatric participants. Another 186 studies were excluded because they were review articles, all participants were blind, had jet lag, were night shift workers, experienced premenstrual symptoms, were pregnant, were menopausal, had Parkinson's disease, or were nonhuman subjects. 187 full-text articles were reviewed.

## 3 Circadian rhythms

The circadian clock in vertebrates and invertebrates, controlled by gene expression, is responsible for synchronizing behavior and bodily functions to a daily rhythm (Patke et al., 2019). This intrinsic rhythm regulates several autonomous molecular oscillators, the external manifestation of which are eating behavior, body temperature, sleep-wakefulness, and hormonal secretion including cortisol, melatonin, thyroid-stimulating hormone, and prolactin (Shechter and Boivin, 2010). Melatonin is one of the most frequently and reliably measured circadian markers. In health, sleep propensity increases approximately 2 h after melatonin secretion rises and these levels remain high in nighttime darkness (Cajochen et al., 2003; Lewy, 2007; Zisapel, 2018).

The central clock in the mammal brain is called the suprachiasmatic nucleus (SCN), comprised of a network of neurons and glia which communicate via neurotransmitters to downstream peripheral clocks (Patke et al., 2019). SCN neurons show spontaneous neuropeptide secretion and cell membrane potential alteration which follows circadian rhythmicity (Patke et al., 2019). The interaction between environmental cues and central clock regulation in humans is still not well understood; however, environmental cues (called zeitgebers) help synchronize the biological clock to the 24 h light-dark cycle in a process called entrainment (Patke et al., 2019). Entrainment is crucial in setting phase-appropriateness of many behaviors.

### 3.1 Core body temperature

The circadian rhythm of core body temperature (CBT) is determined both by changes in heat production and loss. Heat production is phase advanced compared to heat loss (Krauchi, 2007; Kräuchi, 2002) and, in those who are daytime-active, sleep is initiated following a decline in CBT in the evening and ends as CBT rises from its minimum in the morning (Aschoff, 1983; Van Someren, 2006). Peripheral vasodilation, which can improve heat transportation, is partly controlled by melatonin (Kräuchi et al., 2000; Krauchi et al., 1997). Wireless wrist band thermometers have been used to measure this circadian biomarker (Harfmann et al., 2017; Sarabia et al., 2008). Other options include headband thermometers, measurement in the gut through telemetry, or from the axilla. However, the gold standard to measure core body temperature is rectal thermometry, and studies have shown differences between rectal thermometry and other techniques (Edwards et al., 2002; Shann and Mackenzie, 1996).

### 3.2 Rest activity rhythm

Daily body movement and bed rest follow a 24-h circadian rhythm which can be measured by actigraphy (Pollak et al., 2001). Measurements from these devices have been shown to reliably correlate with melatonin and body temperature rhythms (Ancoli-Israel et al., 2003).

## 4 Sleep architecture

Sleep wake cycle is part of the circadian rhythm. Sleep is broadly categorized into Rapid Eye Movement (REM) and non-Rapid Eye Movement (non-REM) stages.

Sleep stages can be measured by Electroencephalography (EEG). Normal sleep architecture at the beginning of sleep is characterized by relatively quick transitions from wakefulness (characterized by beta waves (16–32Hz) (Hsu et al., 2017)), to drowsiness (characterized by alpha waves (8–12 Hz) (Foster et al., 2017)), to N1, N2 and N3 (Table 1). The following definitions are used in relation to sleep efficiency: Sleep Onset Latency (SOL) refers to the time it takes for one to transition from a wakeful state, Slow Wave Sleep (SWS) refers to N3 sleep, characterized by delta waves, Total Sleep Time (TST) as the name suggests, is the entire time in any non-REM or REM stage, Wake time After Sleep Onset (WASO) is the time awake after sleep onset and before termination of sleep.

**Table 1:** Sleep stages and EEG wave type characteristics (Al-Salman et al., 2019; Rodenbeck et al., 2006).

Stage	Wave Type	Frequency
N1	Vertex sharp waves	3–6 Hz
N2	Spindles and K complexes	11–16 Hz and 8–16 Hz respectively
N3	Delta	0–3 Hz
REM	Sawtooth Theta and low voltage waves at random intervals	Varies

For SOL, lower is typically better. Increased TST is also typically a positive indicator of sleep, though one must take into consideration WASO and quality of sleep (how much is SWS and REM). Increased WASO indicates potential fragmentation of sleep, and restlessness during the night.

## 5 Screening considerations

In this section, the effect of drugs and alcohol, shift work, and menstrual cycle phase on circadian rhythm is reviewed, and suggestions are provided about how each one could be addressed in the screening process for a circadian study. Whenever possible, a range of options are provided from the “most strict” to “moderate” so that the investigators can select the best balance between the aims and methods. However, when a criterion is necessary for a reliable study, it is simply labeled “recommended”.

### 5.1 Drug and alcohol considerations

#### 5.1.1 Caffeine

The stimulant effect of caffeine is in part mediated by a robust antagonism of all four adenosine receptor subtypes in the brain (A_1_, A_2a_, A_2B_ and A_3_), and especially the adenosine A_2a_ (ADORA2A) receptor, which is thought to be directly responsible for inducing wakefulness (Evans and Battisti, 2018). This is compounded with caffeine's affinity for cardiac adenosine A_1_ receptors, leading to increased ionotropy, and indirect release of catecholamines which in turn can initiate a fight or flight state (Evans and Battisti, 2018). Caffeine antagonizes A_1_ receptors in the brain leading to a reduction in cAMP production (Burke et al., 2015). cAMP is a key secondary messenger involved in coordinating circadian rhythm in mammals (Burke et al., 2015) (O'Neill et al., 2008). Other mechanisms include inhibition of phosphodiesterases inducing cAMP-dependent signaling and intracellular Ca^2+^ release, which affects presynaptic GABA release in SCN (Chen & van den Pol, 1997). Finally, caffeine can suppress melatonin levels in humans. (Wright, Badia, Myers, Plenzler, & Hakel, 1997).

When administered 30 min before bedtime, caffeine produces a dose-dependent decrease in REM latency, and decreases in deep slow wave sleep (Karacan et al., 1976). Even administration of 200 mg of caffeine (the equivalent of almost 2 cups of brewed coffee) in the morning (before 8am) can decrease sleep efficiency, while subjective sleep quality remains unchanged (Landolt et al., 1995; Rahman et al., 2017).Moreover caffeine consumption increases CBT (McHill et al., 2014) and stimulates locomotion (El Yacoubi et al., 2000). In this way, caffeine use influences a diverse number circadian rhythm biomarkers.

The typical course of caffeine withdrawal begins 12–24 h after abstinence, and peaks at roughly 1–4 days with continued abstinence. The duration of withdrawal is typically 2–9 days (Juliano and Griffiths, 2004). Withdrawal symptoms can occur after chronic use of as little as 100 mg of caffeine per day, with severity of symptoms correlating to the quantity of daily consumption (Juliano and Griffiths, 2004). Withdrawal from caffeine also produces EEG abnormalities such as increased theta and beta wave power in acute abstinence (Sigmon et al., 2009).

For these reasons, many studies (Table 2) prohibit caffeine consumption ranging from 3 h to 3 weeks prior to the study. While there were no explicit reasons given behind the longer duration of exclusions, it is likely that the investigators wanted to ensure there were no withdrawal symptoms that could potentially affect circadian rhythms. **Based on the length of caffeine withdrawal, the most strict recommendation is that participants remain abstinent from any caffeine containing substances for at least nine days before the beginning of their circadian study and throughout the study [Most Strict]. However, an alternative is another commonly used requirement** (Table 2) **to abstain from caffeine within three days (72 h) of the circadian study and throughout the study. [Strict]** Other studies required abstinence or restricted caffeine intake (200–500 mg/day) for certain duration before the study up to 3 weeks and throughout the study. **However, based on most frequently used method in the previous literature** (Table 2) **a moderately strict requirement, which may involve less change from daily habits, is a restriction of no more than 300 mg caffeine per day for at least nine days before the beginning of the circadian study and throughout the study [Moderate].** An additional consideration is the timing of the caffeine intake. Three studies in Table 2 restricted the timing of caffeine to earlier in the day. This can be used in combination with the moderate restriction. If significantly more caffeine is normally ingested, it is helpful to taper intake to avoid uncomfortable symptoms like migraine headaches.

**Table 2:** **Commonly used Exclusion Criteria regarding Exercise, Medication and other Substances:** When multiple studies with similar authorship used the same methodology, a representative publication from each is selected. Duration of abstinence of the activity or substance prior to the study is indicated. (All of these restrictions remained during the study.) “Habitually” refers to the time period until the study. Dosage restrictions and durations are listed. A listed duration without dosage specification means that complete abstinence was required. If any history of this activity or substance would exclude a subject, "any, past or current" was listed. “Any” means that the study did not include subjects who were taking the listed substances. This does not specify duration or type. DOS indicates the substance or activity must be abstained from for the duration of the study.

Citation	Abstinence from excessive exercise	Abstinence from caffeine	Abstinence from alcohol	Abstinence from nicotine	Exclusion for drugs of abuse	Exclusion for psychotropic medications	Other Medications (Other than psychotropic) excluded?	Questionnaires or lab work used for screening
Almeneessier, A. S. et al., (Almeneessier et al., 2017)			any	smokers excluded		any	any	
Baron, K. G. et al., (Baron et al., 2017)		≤300 mg/day habitually	no abuse	smokers excluded	any	any	beta blockers	
Bogdan, A. et al., (Bogdan et al., 2001)				smokers excluded		any	any	
Bojkowski, C. J. et al., (Bojkowski et al., 1987)		12 h	12 h		any			
Burgess, H. J. et al., (Burgess et al., 2015)		≤300 mg/day habitually	≤2 drinks/day habitually	nicotine-free on utox		any	any	utox
Burgess, H. J. et al. (Burgess et al., 2017)		≤300 mg/day habitually	≤14 drinks/week habitually	DOS	DOS			utox
Burke T. M et al. (Burke et al., 2013)	3 days	2 weeks	2 days				OTC, supplements 2 weeks	utox, alcohol breath test
Cajochen et al., 1999 (Cajochen et al., 1999)		2 weeks	2 weeks	2 weeks	2 weeks	2 weeks	any for 2 weeks	utox
Chang et al.		3 weeks	3 weeks	3 weeks	3 weeks	3 weeks	any for 3 weeks	utox
Chellappa, S. L. et al., (Chellappa et al., 2011)		≤1 cup/day habitually	≤5 per week habitually	smokers excluded	any	any	any	utox
Crowley, S. J. et al., (Crowley and Eastman, 2013)		not excessive habitually, none on day of assessment	no abuse, none on day of assessment	DOS	DOS	DOS	any except contraceptive	utox, alcohol breath test
Cuesta M et al. (Cuesta et al., 2015)	No inclusion/exclusion in this category							
Cugini, P. et al., (Cugini et al., 2001)	DOS	not excessive DOS	no abuse				any “spurious” medications 5 days prior	
Danilenko, K. V. et al., (Danilenko et al., 2000)				smokers excluded	any	any	any	
Davies SK et al. (Davies et al., 2014)	72 h	72 h	72 h				NSAIDs 72 h	
Dewan, K. et al., (Dewan et al., 2011)		≤200 mg/day habitually		any tobacco excluded		any		
Figueiro, M. G. et al., (Figueiro et al., 2011)		from 10AM day of				antidepressants	MID, sleep medications, blood pressure medications, beta blockers	
Gabel, V. et al., (Gabel et al., 2013)		1 week	1 week	smokers excluded	any	any	any	
Gann et al., 2004 (Gann et al., 2004)			14 days			7 days		
Gimenez, M. C. et al., (Gimenez, Beersma, Bollen, van der Linden and Gordijn, 2014)				smokers excluded				
Goel, N. (Goel, 2006)	DOS	DOS	DOS					
Gonnissen, H. K. et al., (Gonnissen et al., 2012)	avoid before sleep	avoid before sleep	moderate consumers only	smokers excluded		any	any	
Gorfine, T. et al., (Gorfine and Zisapel, 2009)		DOS	24 h					
Graham, C. et al., (Graham et al., 2001)		6 h before lights off	24 h					
Hajak, G. et al., (Hajak et al., 1996)			24 h	24 h		any	any	
Hallam, K. T. et al., (Hallam et al., 2005)		≤1.4 ± 1.2 standard drinks daily habitually	≤4.5 ± 0.5 standard drinks per week habitually					
Hebert, M. et al., (Hebert et al., 1999)		only allowed in first hour after waking	DOS					
Heo, J. Y. et al., (Heo et al., 2017)			no abuse		any	current use		
Hernandez, C. et al., (Hernandez et al., 2007)			no active abuse			tricyclic antidepressants	MID, anti-epileptics	
Ho Mien, I. et al., (Ho Mien et al., 2014)		1 week	1 week	smokers excluded				
Howatson, G. et al., (Howatson et al., 2012)	No inclusion/exclusion in this category							
Jean-Louis, G. et al., (Jean-Louis et al., 2000)								
Kim, S. J. et al., (Kim et al., 2014)		≤200 mg/day habitually		≤3 cigarettes per week habitually		any	beta blockers, calcium antagonists	
Kozaki, T. et al., (Kozaki et al., 2008)		3 h	1 day	3 h				
Krauchi, K. et al., (Krauchi et al., 2002)				smokers excluded	any	any	any	
Kubota, T. et al., (Kubota et al., 2002)						any		
Lasko, T. A. et al., (Lasko et al., 1999)		5 h	5 h			any	any except contraceptive, NSAIDs for 24 h	
Leproult, R. et al., (Leproult, Van Onderbergen, L'Hermite-Baleriaux, Van Cauter and Copinschi, 2005)			no abuse		any			
Liebrich, L. S. et al., (Liebrich et al., 2014)			no regular or excessive use before and complete abstinence on the assessment day		any	any	“regular medication”	substance questionnaires
Luboshitzky, R. et al., (Luboshitzky et al., 2002)		DOS	DOS	DOS		any	any	
Lushington, K. et al., (Lushington et al., 2002)							prescription, except contraceptive	
Mayeda, A. et al., (Mayeda et al., 1998)			24 h				any prescription (for 2 weeks) and OTC (for 1 week) drugs	
Morera, A. L. et al., (Morera et al., 2009)		12 h	12 h	smokers excluded	any			
Munch, M. et al., (Munch et al., 2006)		≤1 cup/day 1 week before	≤5 per week 1 week before	smokers excluded				
Najjar, R. P. et al., (Najjar and Zeitzer, 2016)			no abuse					Alcohol Use Disorders Identification Test
Oba, S. et al., (Oba et al., 2008)						any	sleep disorder medications	
Paul, M. A. et al., (Paul et al., 2011)		DOS	24 h	smokers excluded			beta blockers	
Pires, M. L. et al., (Pires et al., 2001)				smokers excluded		72 h	“regular medication”	
Flausino N H et al., (Flausino et al., 2012)	6 months	<300 mg/day habitually	≤3 per day habitually	smokers excluded	any habitually	1 month	analgesics, hypnotics, stimulants 1 month	
Rajaratnam, S. M. et al., (Rajaratnam et al., 2004)		≤300 mg/day habitually and none 1 week before	≤90 mg average daily habitually, and none 1 day before through DOS	smokers excluded	any			
Rao, M. L. et al., (Rao et al., 1996)	“days prior”	not excessive habitually	no abuse, and none 1 day before	exclude habitual and none 1 day before	any			
Redwine, L. et al., (Redwine et al., 2000)						any	beta blockers, PG inhibitors, NSAIDs	
Revell, V. L. et al., (Revell et al., 2012)		≤300 mg/day habitually	≤2 drinks/day habitually	smokers excluded	any	any	any prescription	
Richardson, G. S. et al., (Richardson et al., 2008)		≤500 mg/day habitually	no abuse	≤3 cigarettes/day habitually	any		any medications affecting sleep/wake	
Ruger, M. et al., (Ruger et al., 2006)				smokers excluded		any	any	
Rupp, Tracy L. et al., (Rupp et al., 2007)						any	any sleep/wake/sleepiness influencing drugs	
Selmaoui, B. et al., (Selmaoui et al., 1996)		24 h	24 h	smokers excluded				
Skene, D. J. et al. (Skene et al., 2018)		1 week	1 week					
Smith, M. R. et al., (Smith et al., 2009)		≤300 mg/day habitually		smokers excluded	any	any	any prescription	utox
Takasu, N. N. et al., (Takasu et al., 2006)								
Voultsios, A. et al., (Voultsios et al., 1997)		24 h	24 h	smokers excluded			beta blockers, benzodiazepines, MID	
Warman, V. L. et al., (Warman et al., 2003)					any		beta blockers, benzodiazepines, MID	
Wehr, T. A. et al., (Wehr et al., 2001)						3 weeks	any 3 weeks	
Wirz-Justice, A. et al., (Wirz-Justice et al., 2002)	3 days	none allowed in afternoon for 3 days before	3 days	smokers excluded		any	any	
Wright, H. R. et al., (H. R. Wright, Lack and Partridge, 2001)		None habitually	none habitually				habitual hypnotic med user	
Wright, K. P., Jr. er al, (K. P. Wright, Jr. et al., 1997)		Taper to 50–200 mg daily for 1 week, and none allowed 24 h before and DOS	24 h	any tobacco excluded		any	any, NSAIDs for 72 h	
Zeitzer J. M. (Zeitzer et al., 2014)			no abuse	smokers excluded		antidepressants	MID, sleep altering medications, antihistamines, benzodiazepines	
Zhu, Y et al., (Zhu et al., 2013)		≤360 mg/day habitually		≤10 cigarettes/day DOS	any		“most prescription”	
Zimmermann, R. C. et al., (Zimmermann et al., 1993)								utox, blood and urine screen

Melatonin Influencing Drugs (MID), Non-Steroidal Anti Inflammatory Drugs (NSAID), over the counter (OTC), duration of study (DOS), Urine Toxicology screen (Utox).

#### 5.1.2 Alcohol

Animal studies have shown that alcohol can interfere with circadian rhythm by blunting circadian locomotor response to both photic and non-photic cues (Brager et al., 2010, 2011). These effects appear more prominent among adult compared to adolescent mice (Ruby et al., 2017). In addition to these actions, alcohol has been shown to affect melatonin levels. 140–190 min after vodka consumption (0.54 g/kg in men and 0.49 g/kg in women), melatonin was found to decrease 15% and 19% respectively (Rupp et al., 2007). The mechanism may be through norepinephrine. Alcohol is known to alter norepinephrine release for hours after acute intoxication and release of melatonin by the pineal gland is mitigated by norepinephrine (Drijfhout, van der Linde, Kooi, Grol and Westerink, 1996; Ekman et al., 1993) A study by Stevens et al. found that urinary 6-sulfatoxymelatonin, a major metabolite of melatonin, was reduced as long as 24 h after alcohol consumption in women. This was surprising given that the participants did not have a measurable blood alcohol level, but indicates that alcohol may have residual effects on pineal melatonin release. (Stevens et al., 2000).

Alcohol mediates alterations in sleep physiology and sleep architecture via agonism and antagonism of a number of different neurotransmitters (Garcia and Salloum, 2015). Additionally, alcohol increases susceptibility to apneic episodes in those prone to sleep apnea (Simou et al., 2018). Alcohol consumption has been found to reduce sleep latency at all dosages, and increase slow wave sleep resulting in increases in deep sleep during the first half of the night, and decreased quality in the second half. REM sleep was found to be suppressed in the first half of the night only at higher doses (≥0.75 mg/kg of EtOH, which is roughly equivalent to 4 standard alcoholic beverages), with a rebound prolongation of total REM during periods of abstinence (Ebrahim et al., 2013).

During the acute withdrawal period from alcohol, which typically lasts less than one week (Brower, 2015), decreases in total time asleep are observed as are decreases in slow wave sleep, and increases in total time in REM sleep. Residual effects on sleep have even been found during subacute withdrawal in patients with alcoholism, which can last up to a month after abstinence (Brower, 2003, 2015). Circadian phase advance of body temperature, sleep and hormone secretion have also been observed as a consequence of alcohol withdrawal (Rosenwasser, 2001).

Due to the above, several studies have excluded Alcohol Use Disorder (AUD) among their participants and prohibited alcohol consumption between 5 h and 3 weeks before the beginning of their proposed sleep studies (Table 2). **It is recommended that participants who meet criteria for AUD under the DSM-5 be excluded from circadian rhythm studies [Recommended]. Moreover, based on the above-mentioned biological evidence suggesting the longest lasting impairment in circadian biomarkers (including sleep) during alcohol withdrawal phase is one month, the most strict guideline would keep all participants abstinent from alcohol for 1 month (4 weeks) before measuring any circadian rhythm [Most Strict]. Across the literature examined** (Table 2)**, the most frequently used time required for alcohol abstinence was 24 h before a study assessment (which corresponds to the acute intoxication on melatonin metabolite levels); therefore this is the least strict recommendation (along with the AUD exclusion) [Moderate].**

Although there is no clear scientific guideline on limits for reasonable alcohol consumption, researchers who wish to control the participants’ alcohol intake during the study can add that participants should consume no more than 5 drinks per week (the most frequently used weekly cap, Table 2) for four weeks (subacute withdrawal period) before the study and throughout the study [Strict].

#### 5.1.3 Nicotine

Daily nicotine injections can entrain circadian rhythm for locomotor, drinking behavior, body temperature and melatonin phase in rats (Ferguson et al., 1999; Gillman et al., 2008; Pelissier et al., 1998). One possible explanation for the effect of nicotine could be the role of nicotinic receptors in melatonin secretion from the SCN (Ferguson et al., 1999). Moreover both nicotine intoxication and withdrawal disrupt both subjective and objective measures of sleep quality in humans. Like other stimulants, intoxication with nicotine delays sleep onset and decreases total sleep time (Jaehne et al., 2009). Nicotine intoxication was also found to decrease slow wave sleep, and increase subjective reports of sleep disturbances (Jaehne et al., 2009). Chronic smokers have almost double the risk of sleep difficulties, though this may be confounded by the fact that many smokers have comorbid mood disorders (Jaehne et al., 2009). Nicotine withdrawal, which begins 4–24 h after abstinence, and can last up to 4 weeks, also leads to subjective and objective impairments in sleep (Jaehne et al., 2009).

Similar to alcohol and caffeine, of the studies that restricted nicotine, the restriction was between 3 h and 3 weeks prior to the study initiation (Table 2) though some studies excluded daily nicotine users due to the difficulty of quitting and potential to relapse. **Due to the effects of smoking on circadian rhythms and objective impairment in circadian sleep pattern after nicotine administration, the majority of studies exclude daily smokers; this is the most stringent recommendation [Most Strict]. If including smokers, based on withdrawal study findings, abstinence from nicotine should occur from 4 weeks before the study and throughout the study [Strict].** (Questionnaires like Fagerstrom Test for Nicotine Dependence (FTND) that assess physical dependence on nicotine smoking may be helpful in assessing if this abstinence is possible (Pomerleau et al., 1989).) **Since it may be challenging for daily tobacco smokers to stop smoking completely, a guideline that allows mild daily tobacco smoking could be helpful. Based on the few studies allowing daily nicotine users, a less stringent recommendation (though the most conservative of these studies) is a daily nicotine cap of three cigarettes from 24 h before the study and throughout the study [Moderate]** (Table 2)**.** Cotinine tests by either blood, saliva or urine can be used to ensure adherence, as they provide an accurate and reliable marker of nicotine use (Florescu et al., 2009). The half-life of cotinine is about 20 h and levels are typically positive up to a week after tobacco consumption (Florescu et al., 2009).

#### 5.1.4 Cannabis

The compound delta-9-tetrahydrocannabinol (THC) mediates the effect that cannabis has on sleep primarily through its partial agonism at cannabinoid type 1 (CB_1_) receptors, which are diffusely located in the frontal cortex, cerebellum and basal ganglia (Schierenbeck et al., 2008). Functionally, CB_1_ receptors in the SCN can interfere with light entrained locomotor circadian oscillation, via GABA release attenuation (Acuna-Goycolea, Obrietan, & van den Pol, 2010). Intravenous and intraventricular administration of THC leads to a dose dependent decrease in mouse rectal temperature (Fitton and Pertwee, 1982). The mechanism for THC induced core body hypothermia is controversial. However, it has been suggested that the reduction in skin temperature is likely due to vasoconstrictive effects of higher doses of THC and reactive vasoconstriction to decreased core body temperature in lower doses (Smirnov and Kiyatkin, 2008).

Preliminary data have shown administration (smoking) of THC mostly led to increased melatonin levels in blood measured at three time points (20, 60 and 120 min) after smoking. In another study, pineal gland cell cultures treated with cannabinol metabolite showed reduced sensitivity to noradrenaline induced melatonin secretion (Koch et al., 2006). This evidence suggests a role for THC in melatonin biosynthesis (Lissoni et al., 1986).

Acute use of cannabis has been shown to decrease sleep latency as well as Wake After Sleep Onset (WASO), with decreased Rapid Eye Movement (REM) sleep, and increased Slow Wave Sleep (SWS) (Angarita et al., 2016). Conversely, chronic users gain a tolerance to the soporific effect of cannabis, with SWS normalizing to normal or baseline levels, while REM sleep remains decreased (Angarita et al., 2016).

Withdrawal from cannabis is known to cause increased SOL and WASO, with increases in REM, and decreases in SWS (Schierenbeck et al., 2008). While one study indicates that sleep difficulties return to baseline in chronic cannabis users roughly two weeks after abstinence (Budney et al., 2003), others observed fluctuations in sleep quality as long as 45 days after abstinence from chronic cannabis use, that could be in part because of resurfacing pre-existing sleep difficulties (Bonn-Miller et al., 2014; Budney et al., 2003).

No studies had specific recommendations for cannabis use. **Based on the above-mentioned finding showing continued sleep circadian rhythm disturbance as late as 45 days after abstinence from chronic use, it is recommended to exclude chronic cannabis users from circadian rhythm studies [Recommendation]. In the absence of a biologically justified cut-off for cannabis abstinence, many (J. F. Duffy, Kronauer, R.E., and Czeisler, C.A, 1996;** Gronfier et al., 2004; Rahman et al., 2017; Shanahan et al., 1999**) studies only include a single “drugs of abuse” category and exclude any past or current users, which is the most strict recommendation [Most Strict]. A moderately strict approach, based on a single study that allowed cannabis users** (Cajochen et al., 1999)**, would be to exclude chronic users and require occasional cannabis users to abstain for two weeks [Moderate].**

#### 5.1.5 NSAIDs

NSAIDs can disrupt circadian biomarkers (like body temperature and melatonin level) and alter normal sleep physiology measured by EEG as a consequence of inhibiting prostaglandin biosynthesis (Murphy et al., 1994). Prostaglandin (PG) D2 is produced in the preoptic area of the hypothalamus, and aids in inducing sleep onset. PG D2 in CNS prolongs the thermogenic effect of PG E2 by increasing PG E2 availability (Gao et al., 2009). Also, tryptophan is the physiological precursor of melatonin synthesis, and aspirin is thought to induce a surge in serum free tryptophan level. Further, several cytokines play a role in the circadian rhythmicity of gene expression and SCN function. (Comas et al., 2017) Because NSAIDs including aspirin can suppress synthesis of various cytokines, they can alter normal sleep physiology and circadian physiology (Hayaishi, 1991; Murphy et al., 1994).

The recommendations for NSAIDs in previous studies, based on restrictions for over the counter or prescription drugs, or specific recommendation for NSAIDs, include prohibiting NSAIDs use for 1 day to 3 weeks before the study period (Table 2). **Based on the most frequently used cutoff in the literature, three weeks abstinence from NSAIDs would be the most strict criterion [Most Strict]. Common NSAIDs such as ibuprofen and indomethacin have half-lives of roughly 6 h** (Brooks and Day, 1991)**, and aspirin's half-life is roughly 15**–**20 min** (Eikelboom et al., 2012)**. According to this, participants who use NSAIDs should not use this medication for roughly 24 h prior to study, as this is roughly 4**–**5 half lives of those most commonly used [Moderate].** However, investigators should consider the reason for NSAID use as rebound pain might disturb sleep acutely or affect study compliance.

#### 5.1.6 Other drugs of abuse

Cocaine and opioids including heroin are some of the most commonly abused substances. An animal study has shown morphine administration can alter gene expression in the rat SCN (Pačesová et al., 2015). Also cocaine can phase shift circadian rhythms in rats via serotonin transporter inhibition in the SCN (Prosser et al., 2014). Like the other drugs of abuse, cocaine and opioids can also disturb circadian rhythms. However, because of difficulty abstaining from these drugs, those who use these substances should generally be excluded from circadian rhythm studies **[Recommended]** (Table 2)**.**

### 5.2 Sleep routines

Circadian rhythms can be altered by changes in the sleep-wake cycle (J. E. Duffy and Dijk, 2002), due in large part to the phase-dependent impact of light on circadian timing (Phillips et al., 2017). For this reason, a number of the studies required participants to maintain a consistent sleep routine (regular timing and duration of time spent in bed in the dark) in the weeks before the study. In addition, given that many individuals chronically restrict their sleep, and sleep loss may impact some aspects of circadian rhythmicity, one study required participants to spend 8–10 h per night in bed before study to ensure the participants are not sleep-deprived (Stothard et al., 2017). The duration of regular sleep pattern required before measuring circadian rhythm ranged from 1 day to 3 weeks (Table 3). In the absence of studies investigating the possible effect of different lengths of regular sleep time on subsequent circadian biomarkers, and **based on the literature, the most conservative recommendation is for investigators to monitor sleep routine (each participant's personal bed and wake time** (Cain et al., 2010)**) for three weeks before circadian assessment to ensure a regular sleep pattern [Most Strict]**. **However, as the most frequently used cut off for regular sleep/wake time before studying circadian rhythm is one week, one week regular bed time monitoring will provide a moderately strict and less cumbersome cutoff consistent with the previous literature [Moderate].** Sleep schedule and sleep pattern adherence consistency can be monitored via actigraphy, as sensors are relatively inexpensive, accurate, and noninvasive (Walia and Mehra, 2019). Table 3 provides a range of other methods which can be used alternatively.

**Table 3:** **Sleep Profile and Methods of analysis:** When multiple studies with similar authorship used the same methodology, a representative publication from each is selected. If shift work, night shifts or travel across time zone were used as exclusion criteria, the duration of time the participant needed to be free of these activities was listed. If participants with sleep disorders or abnormalities were excluded, the method of screening for this was listed. The duration of required regular sleep schedule and the method of monitoring this are also included.

Citation	Time frame for exclusion for shift work/night shift	Time frame for exclusion for travel across time zones	Exclusion for sleep disorders or abnormalities (if so, what criteria, mechanism of assessment and/or questionnaires)	Required regular sleep schedule prior to study, if so, for how long	Method of assessment during controlled sleep schedule prior to study			
					Actigraphy	Sleep log/diary	Time-stamped voicemail	
Almeneessier, A. S. et al., (Almeneessier et al., 2017)	current	2 weeks	Yes, MEQ	1 week	yes			
Baron, K. G. et al., (Baron et al., 2017)	6 months	6 months (more than 2 time zones)	Yes, Home sleep monitoring for apnea and Restless Leg Syndrome, PSQI	1 week	yes			
Bogdan, A. et al., (Bogdan et al., 2001)		2 months						
Bojkowski, C. J. et al., (Bojkowski et al., 1987)				2 days				
Burgess, H. J. et al., (Burgess et al., 2015)	2 years	2 months (more than 1 time zone)	Yes, IRLSS, PSQI, BSAQ, ISI	1 week		yes		
Burgess, H. J. et al. (Burgess et al., 2017)	2 months	1 month (more than 1 time zones)	Yes, IRLSS, ICSD2 for delayed sleep phase disorder, BSAQ					
Burke T. M et al. (Burke et al., 2013)	1 year	3 weeks (more than 1 time zones)	Yes, personal history of sleep problems	1 week	yes	yes	yes	
Cajochen et al., 1999 (Cajochen et al., 1999)	3 years	3 months (more than 2 time zones)	Yes, psychological screening questionnaires	2 weeks	yes			
Chang et al. (Chang et al., 2015)	3 years	3 months (more than 1 time zones)	Yes, personal history of sleep problems	3 weeks	yes		yes	
Chellappa, S. L. et al., (Chellappa et al., 2011)	3 months	1 month	Yes, PSQI, MEQ		yes	yes		
Crowley, S. J. et al., (Crowley and Eastman, 2013)	1 month	1 month (more than 3 time zones)	Yes, PSQI, ESS	1 week	yes	yes	yes	
Cuesta M et al. (Cuesta et al., 2015)				2 weeks				
Cugini, P. et al., (Cugini et al., 2001)	Participants had regular social routines with lights on at 06:00 and off at 23:00, breakfast at 08:00 ± 01:00, lunch at 13:00 ± 01:00, and dinner at 20:00 ± 01:00 (timeframe not specified)							
Danilenko, K. V. et al., (Danilenko et al., 2000)	2 months	2 months		1 week		yes		
Davies SK et al. (Davies et al., 2014)			Yes, MEQ, PSQI, ESS	1 week	yes	yes	yes	
Dewan, K. et al., (Dewan et al., 2011)	current	3 months (more than 2 time zones)		3 weeks	yes	yes		
Figueiro, M. G. et al., (Figueiro et al., 2011)			Yes, MCTQ	1 week	yes	yes	yes	
Gabel, V. et al., (Gabel et al., 2013)	3 months	3 months	Yes, PSQI	1 week	yes			
Gann et al. (2004) (Gann et al., 2004)			Yes, apnea-hypopnea index, PLMS					
Gimenez, M. C. et al., (Gimenez et al., 2014)	2 weeks	2 weeks (more than 2 time zones)	Yes, MCTQ	2 weeks	yes	yes		
Goel, N. (Goel, 2006)			Yes, MEQ	1 week		yes	yes	
Gonnissen, H. K. et al., (Gonnissen et al., 2012)				2 days	yes			
Gorfine, T. et al., (Gorfine and Zisapel, 2009)				1 day				
Graham, C. et al., (Graham et al., 2001)	Participants had regular sleep habit, did not work evenings or nights (timeframe not specified)							
Hajak, G. et al., (Hajak et al., 1996)	Participants did not fulfill the criteria of any type of sleep disorder (timeframe not specified)							
Hallam, K. T. et al., (Hallam et al., 2005)	All participants self-report normal sleep parameters with sleep onset between 22:00 and 24:00, average sleep latency 30 min, and normal sleep duration (8 ± 1.5 h) (timeframe not specified)							
Hebert, M. et al., (Hebert et al., 1999)	current	1 month (more than 2 time zones)						
Heo, J. Y. et al., (Heo et al., 2017; Hernandez et al., 2007)	current	3 months (more than 2 time zones)	Yes, PSQI, ESS					
Hernandez, C. et al., (Hernandez et al., 2007)	current	exclude jet lag	Yes, PSG apnea/hypopnea index, ESS					
Ho Mien, I. et al., (Ho Mien et al., 2014)	any, past or current	3 weeks	Yes, MEQ, PSQI	1 week	yes	yes		
Howatson, G. et al., (Howatson et al., 2012)	current		Yes, personal history of sleep problems					
Jean-Louis, G. et al., (Jean-Louis et al., 2000)	Regular bedtimes roughly 23:00 and wake time roughly 7:00							
Kim, S. J. et al., (Kim et al., 2014)			Yes, PSG apnea index, movement arousal index	3 weeks	yes			
Kozaki, T. et al., (Kozaki et al., 2008)			Yes, personal history of sleep problems	5 days				
Krauchi, K. et al., (Krauchi et al., 2002)	1 month	1 month	Yes, Overnight clinical observation for sleep apnea, MEQ	1 week	yes			
Kubota, T. et al., (Kubota et al., 2002)	3 months	3 months	Yes, personal history of sleep problems	1 week	yes	yes		
Lasko, T. A. et al., (Lasko et al., 1999)				1 week				
Leproult, R. et al., (Leproult et al., 2005)	any, past or current	1 month						
Liebrich, L. S. et al., (Liebrich et al., 2014)			Yes, PSQI, ESS, LISST					
Luboshitzky, R. et al., (Luboshitzky et al., 2002)				1 week				
Lushington, K. et al., (Lushington et al., 2002)	1 month	1 month	Yes, MEQ	1 week		yes		
Mayeda, A. et al., (Mayeda et al., 1998)	“No inclusion/exclusion in this category”							
Morera, A. L. et al., (Morera et al., 2009)	any, past or current	1 month	Yes, Clinical diagnosis					
Munch, M. et al., (Munch et al., 2006)			Yes, Questionnaire	1 week	yes	yes		
Najjar, R. P. et al., (Najjar and Zeitzer, 2016)			Yes, PSQI, MEQ	2 weeks	yes			
Oba, S. et al., (Oba et al., 2008)	current							
Paul, M. A. et al., (Paul et al., 2011)			Yes, MEQ	during month in study	yes			
Pires, M. L. et al., (Pires et al., 2001)			Yes, PSG					
Flausino, N.H. et al., (Flausino et al., 2012)	1 month	1 month	Yes, Clinical diagnosis, PSQI, ESS	1 month				
Rajaratnam, S. M. et al., (Rajaratnam et al., 2004)	recent	recent	Yes, PSG during a lab adaptation night	10 days	yes			
Rao, M. L. et al., (Rao et al., 1996)				“days prior”				
Redwine, L. et al., (Redwine et al., 2000)			Yes, Overnight tibial myoclonus and oxygen desaturation recordings	2 weeks		yes		
Revell, V. L. et al., (Revell et al., 2012)	1 month	1 month (more than 2 time zones)		1 week				
Richardson, G. S. et al., (Richardson et al., 2008)	any, past or current	3 or more time zones recently	Yes, apnea-hypopnea index, movement arousal index	1 week		yes		
Ruger, M. et al., (Ruger et al., 2006)	any, past or current	1 month (more than 1 time zones)	Yes, MEQ	1 week				
Rupp, Tracy L. et al., (Rupp et al., 2007)	3 months	3 months (more than 3 time zones)	Yes, previously diagnosed sleep disorder	10 days	yes	yes	yes	
Selmaoui, B. et al., (Selmaoui et al., 1996)	any, past or current	2 months	Yes, Clinical exam					
Skene, D. J. et al. (Skene et al., 2018)	3 months	1 month	Yes, PSG and CSM, PSQI, SDQ, ESS	1 week	yes	yes	yes	
Smith, M. R. et al., (Smith et al., 2009)	3 months	1 month (more than 3 time zones)	Yes, Questionnaire					
Takasu, N. N. et al., (Takasu et al., 2006)	4 weeks	4 weeks						
Voultsios, A. et al., (Voultsios et al., 1997)			Yes, General health questionnaire					
Warman, V. L. et al., (Warman et al., 2003)			Yes, PSQI, MEQ	2 weeks	yes	yes		
Wehr, T. A. et al., (Wehr et al., 2001)				10 days		yes		
Wirz-Justice, A. et al., (Wirz-Justice et al., 2002)	1 month	1 month (more than 2 time zones)						
Wright, H. R. et al., (H. R. Wright et al., 2001)				1 week				
Wright, K. P., Jr. et al., (K. P. Wright, Jr. et al., 1997)				1 week		yes		
Zeitzer J. M. et al., (Zeitzer et al., 2014)			Yes, MEQ, PSQI	2 weeks	yes	yes		
Zhu, Y et al., (Zhu et al., 2013)	any, past or current	1 month (more than 2 time zones)	Yes, Personal history of sleep problems	1 week				
Zimmermann, R. C. et al., (Zimmermann et al., 1993)	“No inclusion/exclusion in this category”							

Abbreviations: Pittsburgh Sleep Quality Index (PSQI), Morning-Eveningness Questionnaire (MEQ), the Epworth Sleepiness Scale (ESS), and the Landecker Inventar fur Schlafstorungen (LISST), Munich Chronotype Questionnaire (MCTQ), Insomnia Severity Index (ISI), Berlin Sleep Apnea Questionnaire (BSAQ), International Restless Legs Syndrome Study Group consensus criteria for restless leg syndrome (IRLSS). Polysomnography (PSG), Composite Scale of Morningness (CSM), Sleep Disorders Questionnaire (SDQ), International Classification of Sleep Disorders (ICSD).

Multiple studies restricted participants from having crossed more than two time zones in 2 weeks–6 months before the study (Table 3) as these clear alterations in sleep schedules could affect circadian rhythms. **In the absence of scientific justification for these limits, based on the literature, the most cautious exclusion is to restrict travel across two or more time zones within six months before the study [Most Strict]. However, majority of the previous studies used 1 month (4 weeks) as a cut off [Moderate].**

Shift work is difficult to operationalize and largely depends on the schedule (fixed vs rotating). It was presumed that fixed night shift work might provide an opportunity to adjust circadian rhythm to the schedule and thus is healthier than rotating shift work (Costa, 2010). However evidence does not support adaptive circadian rhythm adjustment among fixed night shift workers (Folkard, 2008). Studies that addressed the issue of shift work in participants excluded those who have done any shift work in a period of 2 weeks–3 years before the study (Table 3). **As such, the most cautious recommendation is that participants must not have done shift work in the three years prior to the study [Most Strict]. However, the majority of the studies used one month (4 weeks) cut off for night shift work prior to the assessment [Moderate].**

### 5.3 Menstrual cycle considerations

Subjective sleep disturbances have been reported to increase in late luteal phase, and early follicular phase, around the time when women are menstruating (Fiona C. Baker and Driver, 2004; Kravitz et al., 2005). A meta-analysis demonstrated that most studies showed decreases in duration of REM sleep during luteal phase, with increased sleep spindle density, blunted changes in body temperature, but no melatonin phase shifts (F. C. Baker and Lee, 2018). However, increased, decreased or delayed melatonin secretion onset between luteal and follicular phase have all been reported (F. C. Baker and Driver, 2007) and one study comparing circadian rhythms in men and pre-menopausal women found similar timing of melatonin rise relative to sleep, though they did not account for menstrual phase (Cain et al., 2010).

Some studies found no relationship between sleep disturbances and menstrual cycle, and that quality of sleep did not worsen in the premenstrual stage (Li et al., 2015; Van Reen and Kiesner, 2016). This may be related to how disturbances were reported. Studies using objective measures such as actigraphy and EEG show a decrease in sleep efficiency in the week leading up to menstruation (Zheng et al., 2015). Moreover, women with more severe behavioral/emotional premenstrual symptoms (Premenstrual Tension Syndrome/PMTS-Self Rating Scale score ≥14 during late luteal or ≤5 during follicular) have been shown to be sleepier and less alert during late luteal days but not during follicular phase days, independent of nighttime sleep quality (Lamarche et al., 2007). Further, perimenopausal women have alterations in sleep-wake cycles, with sleep latency, fragmentation, and nocturnal awakenings. Yet, it is unclear if this is related to altered melatonin levels, or due to perimenopausal symptoms such as hot flashes, or changes in mood, disrupting sleep (Lord et al., 2014).

Core temperature during the luteal phase is lower than in the follicular phase (Cain et al., 2010), which may mask circadian changes being assessed by temperature. Further, hypoestrogenic states like postmenopause or hormonal contraceptive pills can increase mean body temperature, SWS period and reduce REM latency in women (Czeisler et al., 1992; Driver and Baker, 1998).

Due to the possible effects listed above, **the most stringent recommendation is to enroll women only during the follicular phase of their menstrual cycle by assessment of hormones or self-report [Most Strict]. A less strict approach could only exclude women during pregnancy, peri-menopausal symptoms or premenstrual syndrome [Moderate].**

### 5.4 Age considerations

Several natural circadian rhythms in the human body change by age, including phase advance in body temperature rhythm, melatonin, cortisol, glucose level peak, and insomnia/fragmented sleep (Beattie et al., 2015; Hood and Amir, 2017).

The human eye also may lose its sensitivity to light secondary to reduced lens transparency. Decreased light transmissibility leads to reduced signaling to SCN and less frequent circadian signaling to target organs (Hood and Amir, 2017). On the other hand, other studies have suggested melatonin reduction is not observed in the healthy aging process and is only associated with cognitive impairment and prodromal symptoms of neurodegenerative disorders (Hood and Amir, 2017; Kin et al., 2004). Many of the age-associated pathological processes can lead to circadian rhythm alteration.

**Given the possible effect of age on circadian rhythms, the recommendation is to keep age range as limited as possible.**

## 6 Protocol considerations

In setting up any circadian study, investigators should carefully design their measurement tools to capture circadian variables without external influence (J. E. Duffy and Dijk, 2002). The endogenous circadian variables commonly studied are melatonin (either plasma or salivary), core body temperature and cortisol (plasma or salivary). Oscillatory confounds that affect circadian rhythm are generally categorized as endogenous and environmental (Wirz-Justice, 2007). Dawn and dusk timing (varies by season), quality of light exposure, body posture (flat vs. upright) and behavioral measures such as bedtime are some of the environmental variables. The most accurate endogenous circadian biology is measured when environmental variables are well controlled. For this purpose, investigators traditionally eliminate the effects of environmental factors using the Constant Routine (CR) or forced desynchrony protocol. In these protocols, participants are kept dissociated from any circadian environmental changes, so that any measurement in circadian biomarkers will reflect pure endogenous oscillatory biology (Wirz-Justice, 2007). While these studies have provided invaluable information to the field, many studies will benefit by evaluating participants in a more natural setting. The following *subheadings* will describe each of the most common environmental-behavioral variables and how to control them. We focus on controlling these variables in the sampling of melatonin.

### 6.1 Lighting

Light exposure is known to suppress blood and saliva levels of melatonin via the retinohypothalamic pathway and suprachiasmatic nucleus (Sadun et al., 1984). Bright (>2500 lux) light was found to suppress melatonin levels completely (Arendt, 2005) but even overhead lighting of much lower brightness (100 lux from fluorescent bulbs) can cause significant suppression (Bojkowski et al., 1987; Harada, 2004; Iwamoto et al., 2018). Initially, it was believed melatonin levels decrease linearly in proportion to the light exposure from 700 Lux (Mayeda et al., 1998). However later studies showed, the light intensity-melatonin concentration response pattern depends on previous light exposure (J. E. Duffy and Dijk, 2002) and follows a non-linear relationship (Figueiro et al., 2005; Kozaki et al., 2008).

It has been shown that dimming the light 8 h before habitual sleep time can significantly prolong the melatonin detection duration in the body by removing the melatonin suppressing effect of bright light (Gooley et al., 2011). Multiple studies have validated that the onset of melatonin secretion under dim light (Dim Light Melatonin Onset, or DLMO) is the single most accurate circadian biomarker (Pandi-Perumal et al., 2007). Further, DLMO collected under lab conditions (light <5 lux) and less restrictive home conditions (<50 lux) were highly correlated (r = 0.91) (Burgess et al., 2015) and consistent with DLMO measurements collected under a previously published melatonin measurement guideline which suggests light intensity <30 lux. (Benloucif et al., 2008).

**The most strict guideline would keep participants in dim light (<50 Lux) for 8 h before habitual sleep onset** (Gooley et al., 2011) **[Most Strict]. However, based on melatonin secretion onset, remaining in the dim light for 4 h before habitual sleep onset is a more moderate guideline [Moderate]** (Burgess et al., 2015). For comparison, when reading an e-book, the reader is exposed to roughly 38 lux, versus the approximately 0.7 lux that is reflected from a paper book. However, these values are highly variable and depend on the model of the e-reader device, the light intensity setting, and ambient light (St Hilaire et al., 2012). Certain devices provide settings for light emittance, and all can be checked with a relatively inexpensive photometer.

### 6.2 Sampling melatonin

Traditionally melatonin in blood (via antecubital venous catheter), saliva (cotton swab vs free saliva) or urine metabolites have been used to assess circadian rhythmicity in human neuro-endocrine system. (Benloucif et al., 2008) These three methods have been shown to be equally precise (Mullington et al., 2016). Ease of constant access throughout the night without requiring postural changes or sleep interruption, make the plasma melatonin desirable. Blood drawing from an adjacent room will allow sampling and labeling without interrupting the participant's sleep. A venous catheter from an adjacent room can be kept patent using a heparinized saline pump to prevent clotting. **Reliable blood sampling should be performed at least 2 h after inserting the IV catheter to ensure that adrenergic response to placement will not affect melatonin levels [Recommended]** (Benloucif et al., 2008).

### 6.3 Posture

Posture is an important exogenous factor affecting melatonin release. Melatonin levels rise when changing from a lying down to sitting position (Arendt, 2005; Nathan et al., 1998; Ritz-De Cecco, Jewett, Duffy, Shanahan, & Czeisler, 2001). However, these postural changes in plasma melatonin were noted to be reversible within 10 min (Benloucif et al., 2008) Because of this, **when drawing blood to analyze plasma melatonin levels during a study, it is recommended that participants remain in a stable position (either lying down or sitting) for 10 min prior to the blood draw [Recommended].**

### 6.4 Exercise

Exercise can alter circadian biomarkers. One study found blunted nocturnal melatonin peaks in healthy men with no regular exercise habits following acute, high intensity exercise for 40 min, ending a half an hour before bedtime (Monteleone et al., 1990). However, other studies report no change in nocturnal melatonin level measured 5–30 min following acute exercise compared to baseline among physically active men and women (Elias et al., 1993; Ronkainen et al., 1986). While the reasoning behind the different findings is unknown, possible variability in hydration status could underlie these discrepancies.

Exercise affects sleep physiology diversely, depending on duration of the training, time of day, age, and existence of prior sleep disorders (Chennaoui et al., 2015). Regular exercise has proven beneficial effects on sleep efficiency including increasing SWS and TST and decreasing REM sleep, REM sleep latency, SOL and WASO (Blanco-Centurion and Shiromani, 2006; Brand et al., 2010; Buman et al., 2011; Irwin et al., 2008; Kalak et al., 2012; Kubitz et al., 1996; Lang et al., 2013; Naylor et al., 2000; Oudegeest-Sander et al., 2013; Yang et al., 2012). These changes can persist up to 9 weeks after the exercise (Irwin et al., 2008).

The effect of acute exercise on polysomnographic parameters seems to depend on its proximity to sleep. If the exercise occurs 4–8 h before sleep onset, SOL and WASO may be improved (Kubitz et al., 1996; Youngstedt, O'Connor and Dishman, 1997). However, exercise within 4 h of bedtime may affect SOL and WASO negatively, likely mediated by central nervous system activation and norepinephrine release (Driver and Taylor, 2000; Kubitz et al., 1996; Youngstedt et al., 1997). Moreover exercise induced catabolism can cause increased body temperature (O'Connor et al., 1998).

**Previous circadian rhythm studies have recommended avoiding excessive exercise from 3 days to 6 months before circadian rhythm study** (Table 2)**. Therefore, the most strict recommendation is to avoid any out of routine exercise during the 6 months preceding the circadian biomarkers measurement [Most Strict]. While there is no evidence regarding a regular exercise schedule, the adverse circadian effects of acute (out of routine) exercise appears to last up to 4 h. For this reason, a moderately strict recommendation is that participants avoid physical activity out of their routine at least 4 h before measuring melatonin level [Moderate].** With this recommendation, alterations in circadian melatonin level, body temperature and sleep pattern can be avoided, regardless of whether the participant habitually exercises or not. Compliance can be assessed with actigraphy or onsite monitoring.

### 6.5 Social interactions

Social zeitgebers, including recreational activities, social engagements, timing of meals and entertainment (e.g., television), play a role in entrainment (Monk et al., 1990). In fact, although light is a more powerful zeitgeber, social cues may influence exposure to bright light as well as affect the sleep-wake cycle (Elmore et al., 1994), though these effects may be mediated by chronotype (Korczak et al., 2008). Just as sleep routines are affected by travel across time zones, the difference between biological and social time (including time of work obligations and daylight savings time (Kohyama, 2011)) can lead to “social jetlag” (Kramer and Merrow, 2013; Wittmann et al., 2006) and studies have reported effects of social jetlag on melatonin (for example, in night shift workers (Vieira et al., 2021) and people with discordant sleep on work and free days (Geerdink et al., 2016; Gimenez et al., 2010)).Therefore, like other non-photic zeitgebers (e.g., exercise, food) (Schulz and Steimer, 2009), the effects of social interactions should be monitored.

Based on the above, consistency in sleep-wake cycle, as described in 5.2, before and during the lab visit will likely require limitation of social cues/interactions during regular sleep hours. This should be discussed with participants. We further recommend that studies are not scheduled during or immediately after Daylight Savings Time [Recommended].

Also important to consider is that emotionally-charged conversations and social interactions affect sleep (Vandekerckhove and Wang, 2018) and potentially melatonin. Though melatonin's effects on mood is often studied (Dollins et al., 1993), the relationship could be bidirectional, or indirect. Therefore, consideration should be given to preventing such interactions before melatonin collection and sleep.

### 6.6 Dietary considerations

Short term (two to seven days) calorie restriction (<300 kcal per day) reduces nighttime melatonin release, up to 20% in some cases (Michalsen et al., 2003; Peuhkuri et al., 2012; Rojdmark et al., 1992; Rojdmark and Wetterberg, 1989). However, one study found that during fasting states, there were decreases in the number of arousals during sleep, decreases in periodic leg movements, and a nonsignificant trend toward increased REM sleep despite lower melatonin levels during these states (Michalsen et al., 2003). These findings were associated with increases in subjective measures of sleep quality. The authors attributed these seemingly paradoxical findings to the increases in growth hormone, decreased thyroid hormone, and increased serotonin output (Palmblad et al., 1977; Voderholzer et al., 1998). Although meal timing and calorie content may affect core body temperature and melatonin magnitude, it has not been established that this results in a phase shift of melatonin (Krauchi et al., 2002). Therefore, if melatonin level is the variable of interest, participants should comply with an hourly distributed isocaloric snacks for the duration of the assessment (Krauchi et al., 2002). For all assessments, **participants should avoid dietary restrictions and change in their diets (e.g., intermittent fasting, calorie restricted diet, etc.) from a week before the study [Recommended].**

Dietary amino acids (tryptophan), vitamins and minerals (co-enzymes) and melatonin content of certain food like tomatoes, walnuts, strawberries, barley or other grains may contribute to daytime fluctuation of plasma melatonin (Zeng et al., 2014); however, nighttime consumption of melatonin enriched meals in adults has not been shown to affect night time melatonin. In a study after melatonin enriched milk was served to an elderly population, minimal change in nocturnal melatonin levels and sleep quality was reported (Peuhkuri et al., 2012; Valtonen et al., 2005). However, in this study the investigators did not report the precise time when the milk was served, so it is difficult to speculate whether or not the ingested melatonin half-life would overlap with the measured DLMO (Valtonen et al., 2005). In fact, several factors may be involved in the effects of dietary melatonin on changes in measured melatonin, including type of the food/beverage), age range, time before measurement of melatonin and plasma melatonin versus urinary metabolites (Kennaway, 2017).

Since the half-life of plasma melatonin is short (<60 min) (Peuhkuri et al., 2012), and in the absence of knowing the length of time that ingested melatonin may affect measured melatonin, the most conservative suggestion is **to avoid food or beverages containing melatonin/precursors within 60 min (plasma melatonin half-life) before the melatonin assessment [Most Strict]. However, given that the data is equivocal an argument can be made for not restricting these foods [Moderate].**

## 7 Alternatives and innovation in circadian rhythm biomarkers

Traditional circadian rhythm biomarkers, including hormones like melatonin and body temperature rhythms, are the cornerstone in evaluating circadian biology. However recent discoveries and technological advancements have introduced additional exciting techniques for assessing circadian biology on cellular and molecular levels without requiring an extended stay in the laboratory. For example, skin fibroblasts transfected with bioluminescent or fluorescent reporters can provide a readout of circadian gene expression (Saini et al., 2015). Quantification of circadian rhythm *in vitro* in this way significantly reduces experimental cost and time requirements, as well as laboratory animal use. This technique could also be used for high throughput screening of genetic or exogenous effects on circadian rhythm (Fang et al., 2017). Further, it could be extended to primary cell culture from peripheral organs for tissue-specific circadian information that may relate to disease state (e.g., human pancreatic islet circadian rhythm in diabetes) (Saini et al., 2015). While there are significant advantages to such techniques, further research is needed. For example, though circadian estimates from skin fibroblasts were shown to be reliable, they may not recapitulate traditional melatonin assessments (Hasan et al., 2012).

Another consideration in circadian research is that neuroreceptor circadian oscillation may underlie behavior and could be disrupted in mood disorders. Metabotropic glutamate receptor-5 (mGluR5) availability increases 10% during sleep phase in rats (Elmenhorst et al., 2016) and a circadian pattern of mGluR5 availability has been demonstrated in healthy individuals (DeLorenzo et al., 2017). Also circadian variability in extracellular glutamate levels in the suprachiasmatic nucleus (SCN) of rat has been shown (Honma et al., 1996). mGluR5 availability, extracellular glutamate concentration, and, potentially circadian rhythm in other neurotransmitters, provide exciting markers of diurnal changes. However their interaction in physiological and pathological states of circadian rhythm need to be further investigated.

## 8 Conclusion

In order to improve our understanding of circadian rhythms and their role in disease states, there is an increasing need to perform well-controlled circadian studies on humans in a more natural setting than those of the CR or forced desynchrony studies. Above are recommendations for these studies based on innovative and seminal circadian research performed to date, from basic science to clinical trials. The recommendations provide a range of guidelines that will allow investigators to consider study options and make well-informed decisions about their screening procedures and protocol techniques.

## Ethics approval and consent to participate

Not Applicable.

## Consent for publication

Not Applicable.

## Availability of data and materials

Data sharing not applicable to this article as no datasets were generated or analyzed during the current study.

## Declaration of interest

The authors declare that they have no competing interest.

## Funding

None.

## Summary of recommendations

### Screening

#### Caffeine

- Most Strict: abstain from caffeine from nine days before study.
  o Based on the length of caffeine withdrawal
- Strict: abstain from caffeine from three days before the study
  o Commonly used alternative
- Moderate: ≤300 mg daily from nine days before study.
  o Most frequently used in the literature (Table 2), and based on the length of caffeine withdrawal
- For the strict and moderate option, timing of caffeine should be considered.

#### Alcohol

- Recommendation: exclude those who meet criteria for alcohol use disorder
- Most Strict: abstain from alcohol from 4 weeks before study
  ○ Based on the longest lasting impairment in circadian biomarkers (including sleep) during alcohol withdrawal phase (subacute withdrawal period)
- Strict: ≤5 drinks per week from 4 weeks before the study and no alcohol 24 h before any study assessment
  o Based on the most frequently used weekly cap in the literature (Table 2), subacute alcohol withdrawal period and acute effect of alcohol on melatonin metabolite levels
- Moderate: no alcohol 24 h before any study assessment.
  o Based on acute effect of alcohol intake on melatonin metabolite levels

#### Nicotine

- Most Strict: exclude daily cigarette smokers
  ○ Based on effect of tobacco smoking on circadian rhythm and the most frequently used exclusion criterion in the literature (Table 2)
- Strict: abstain from cigarettes from one month before study
  o Based on withdrawal period for nicotine
- Moderate: ≤3 cigarettes daily from 24 h before the study.
  o Based on the most stringent of the studies in Table 2 that allowed smoking during the study

#### Cannabis

- Recommendation: exclude chronic cannabis users from circadian studies
  ○ Sleeping difficulties may last beyond 45 days (the longest interval examined) after abstinence in chronic cannabis users
- Most Strict: exclude participants with any past or current cannabis use.
  ○ Lack of precise and biologically reasonable recommendation for cannabis
- Moderate: abstain from cannabis from 2 weeks before study in occasional users
  o Based on a single previous study that allowed occasional cannabis users (Table 2)

#### NSAIDs

- Most Strict: abstain from NSAIDs from 3 weeks before the study
  ○ Based on effects of NSAIDs on sleep and body temperature and most frequently used in the literature (Table 2)
- Moderate: abstain from NSAIDs 24 h before any study assessment.
  o Based on NSAID half-life
- Investigators should consider the reason for NSAID use as rebound pain might disturb sleep acutely or affect study compliance

#### Other drug use

- Recommendation: current substance use should be excluded
  ○ Based on the circadian rhythm disturbance secondary to substance use and difficulty abstaining from the substance use
- Drug use can be assessed during screening for exclusion via Utox
  o Common, accurate and relatively inexpensive objective measure

#### Sleep pattern

- Most Strict: ensure regular sleep pattern for 3 weeks before the study
  ○ Most conservative recommendation in the literature (Table 3)
- Moderate: ensure regular sleep pattern for 1 week
  o Most frequently used in the literature (Table 3)
- Sleep schedules can be assessed during screening via sleep actigraphy
  o Common, accurate and relatively inexpensive objective measure

#### Travel

- Most Strict: avoid travel across two or more time zones from 6 months before the study
  ○ Most conservative exclusion based on the literature (Table 3)
- Moderate: avoid travel across two or more time zones from 1 month before the study
  o Most frequently used in the literature (Table 3)

#### Shift work

- Most Strict: avoid shift work from 3 years before the study
  ○ Most conservative exclusion based on the literature (Table 3)
- Moderate: avoid shift work from one month before the study
  o Most frequently used in the literature (Table 3)

#### Menstruation

- Most Strict: include women only during follicular phase in menstruation.
  o Due to objective and subjective circadian markers disturbance during luteal phase
- Moderate: only exclude pregnant women, women with perimenopausal and premenstrual syndrome
  ○ Due to alterations in sleep-wake cycles in perimenopausal and premenstrual population and that some studies found no relationship between sleep disturbances and menstrual cycle

### Protocol

#### Light

- Most Strict: dim light (<50 Lux) for 8 h before habitual sleep onset
  ○ Can significantly prolong the melatonin detection duration
- Moderate: dim light (<50 Lux) for 4 h before habitual sleep onset
  ○ Melatonin secretion may start about 4 h before sleep onset. DLMO collected under lab conditions (light <5 lux) and less restrictive home conditions (<50 lux) were highly correlated

#### Venipuncture

- Recommendation: if collecting a blood measure of circadian markers, IV placement should be done more than 2 h before first sample is collected
  o To minimize adrenergic response on melatonin levels
- Recommendation: participants should be in a constant posture for 10 min before each blood draw to collect plasma melatonin
  o Plasma redistribution alters plasma melatonin levels, but returns to baseline and remains stable after 10 min of posture change

#### Activity

- Most Strict: avoid any excessive out of routine exercise from 6 months before the study
  ○ The most conservative guideline in the literature (Table 2)
- Moderate: acute physical activity should be avoided 4 h before normal sleep time
  ○ Adverse circadian effects of acute (out of routine) exercise can last up to 4 h

#### Diet

- Recommendation: avoid any change in diet, calorie restriction and intermittent fasting from one week before the study
  ○ Calorie restriction reduces night time melatonin release, meal timing and calorie content may affect core body temperature and melatonin magnitude
- Most Strict: avoid food and beverages containing melatonin for 1 h before assessment
  ○ Based on melatonin half-life
- Moderate: Regular diet without any restriction
  ○ Equivocal data for effect of dietary melatonin on plasma melatonin

## CRediT author statement

Yashar Yousefzadehfard: Methodology, Writing - Original Draft, Visualization. Bennett Wechsler: Writing - Original Draft, Visualization. Christine DeLorenzo: Conceptualization, Writing - Review & Editing, Supervision.

## Declaration of competing interest

The authors declare no conflicts of interest.
